# Cross genome phylogenetic analysis of human and *Drosophila* G protein-coupled receptors: application to functional annotation of orphan receptors

**DOI:** 10.1186/1471-2164-6-106

**Published:** 2005-08-10

**Authors:** Raghu Prasad Rao Metpally, Ramanathan Sowdhamini

**Affiliations:** 1 National Centre for Biological Sciences, Tata Institute of Fundamental Research, UAS-GKVK Campus, Bellary Road, Bangalore 560065, INDIA

## Abstract

**Background:**

The cell-membrane G-protein coupled receptors (GPCRs) are one of the largest known superfamilies and are the main focus of intense pharmaceutical research due to their key role in cell physiology and disease. A large number of putative GPCRs are 'orphans' with no identified natural ligands. The first step in understanding the function of orphan GPCRs is to identify their ligands. Phylogenetic clustering methods were used to elucidate the chemical nature of receptor ligands, which led to the identification of natural ligands for many orphan receptors. We have clustered human and *Drosophila* receptors with known ligands and orphans through cross genome phylogenetic analysis and hypothesized higher relationship of co-clustered members that would ease ligand identification, as related receptors share ligands with similar structure or class.

**Results:**

Cross-genome phylogenetic analyses were performed to identify eight major groups of GPCRs dividing them into 32 clusters of 371 human and 113 *Drosophila* proteins (excluding olfactory, taste and gustatory receptors) and reveal unexpected levels of evolutionary conservation across human and *Drosophila* GPCRs. We also observe that members of human chemokine receptors, involved in immune response, and most of nucleotide-lipid receptors (except opsins) do not have counterparts in *Drosophila*. Similarly, a group of *Drosophila* GPCRs (methuselah receptors), associated in aging, is not present in humans.

**Conclusion:**

Our analysis suggests ligand class association to 52 unknown *Drosophila* receptors and 95 unknown human GPCRs. A higher level of phylogenetic organization was revealed in which clusters with common domain architecture or cellular localization or ligand structure or chemistry or a shared function are evident across human and *Drosophila* genomes. Such analyses will prove valuable for identifying the natural ligands of *Drosophila* and human orphan receptors that can lead to a better understanding of physiological and pathological roles of these receptors.

## Background

G protein-coupled receptors (GPCRs) are one of the largest superfamilies of cellular receptor proteins, generally consisting of seven transmembrane helices (TMH) connected by three extracellular and three cytoplasmic loops of varying lengths. Different GPCRs respond to a wide variety of different external stimuli (light, odorants, peptides, lipids, ions, nucleotides etc) and activate a number of different GTP binding proteins (G proteins), there by initiating a wide spectrum of intracellular responses. GPCRs play important roles in cellular signaling networks involving such processes as neurotransmission, taste, smell, vision, cellular metabolism, differentiation and growth, inflammatory and immune responses and secretion. Abnormalities of signaling by GPCRs are the root cause of disorders that affect most tissues and organs in our body, such as color blindness, thrombosis, restenosis, atherosclerosis, hyper functioning thyroid adenoma and nephrogenic diabetes insipidus and precocious puberty. GPCRs are of major importance to the pharmaceutical industry since they play major roles in the pathogenesis of human diseases and are targets for more than half of the current therapeutic agents on the market [1]. Despite the importance of GPCRs in physiology and diseases, only one high-resolution structure has been solved, that of bovine rhodopsin [2]. A majority of the identified GPCRs are with no known ligand specificity (orphan receptors), which presents a challenge for identifying their native ligands and defining their function.

Characterizing the role of any GPCR involves the identification of both the activating ligand and the activated G protein. A diverse range of procedures have led to the identification of ligands for orphan receptors: (1) identifying relationship between receptor and ligand expression patterns [3], (2) testing tissue extracts in receptor-based functional assays and (3) testing ligands for identified GPCRs on orphan GPCRs with high sequence identity [4] and in some cases randomly evaluating orphan GPCRs against arrayed families of known ligands. The physiological role of these receptors can be well understood by the identification of natural ligands, which further advance the design of pharmacologically active surrogate activators or inhibitors of the GPCRs that have defined native ligands. Strategies described above will be facilitated by better prediction of ligand structure or chemical class of orphan GPCRs.

Proteins similar in sequence often exhibit similar functions. Therefore, sequence homology can be used as a primary criterion for functional screening. This powerful principle can be extended to proteins that are homologous in different species. This has led to the identification of many new novel GPCRs across different species [5]. Many orphan GPCRs are conserved among different species suggesting that they should be active and thus bind novel ligands. This led to the idea that orphan GPCRs could be used as targets to identify their natural ligands and consequently led to the discovery of novel transmitters [6]. Those orphan receptors that share more than 45 percent of sequence identity with the GPCRs with known ligands are very likely to also share common ligands [5]. Often, the direct association of ligand class to orphan receptors is non-trivial by simple BLAST searches even at high sequence identity [7]. The top ranking hits constitute GPCRs from diverse ligand classes (Metpally and Sowdhamini unpublished results) and may not suggest a consensus on possible ligand class to be inferred directly. However, if the sequence identity is below the twilight zone (less than 30 percent), predictions using direct sequence search methods often fail. Phylogenetic tree building has shown that receptors that respond to the same, or similar, agonists often cluster together, even with low sequence identity. For example, most members of the prostanoid receptor subfamily share less than 30 percent amino acid identity, yet these receptors are more like one another than any other GPCR [8]. Phylogenetic clustering methods were used to elucidate the chemical nature of receptor ligands, which led to the identification of natural ligands for many orphan receptors [9-14].

GPCRs were previously classified into distinct families by different groups [14-18]. The classifications would include rhodopsin-like receptors, secretin receptor-like receptors, metabotropic glutamate-like receptors, adhesion-like receptors and frizzled/smoothened-like receptors as proposed by Fredriksson and coworkers [16]; in addition, other groups have proposed two more classes, viz., the fungal pheromone receptor like family and cyclic AMP receptors family [17,18]. These classification schemes were generated mostly from individual genome studies [12,16].

Studies in model organisms and cross-genome comparisons have provided major insights in the general understanding of numerous genes and pathways involved in a wide variety of physiological processes and human diseases [19]. *Drosophila* is a very good model organism owing to the simplicity in the genetic system and a short lifespan enabling the screening of large individuals to identify mutations in new candidate genes that may have human counterparts involved in cellular physiology and diseases [20]. Despite disparity in morphology or phenotype, *Drosophila* shows similarity with humans in developmental and cellular processes like core aspects of cell cycle, signaling pathways, apoptosis, neuronal signaling, cytoskeleton and core proteome (including main protein domains and families) [21]. We, therefore, sought out to adopt *Drosophila* GPCRs to study human gene function using comparative genomics [21-23].

A large number of *Drosophila* GPCRs have no characterized ligands. On the other hand, many human GPCRs are well characterized in their physiology and pharmacology. In this study, we collected a large set of GPCR sequences from human and *Drosophila* genomes and performed cross-genome multiple phylogenetic analyses. Further analysis reveals unexpected levels of similarity between GPCRs of these two species and phylogenetic association could be employed to predict ligands (chemical structure or class and/or functions) for many of *Drosophila* and human orphan receptors.

## Results and discussion

**Figure 1:**
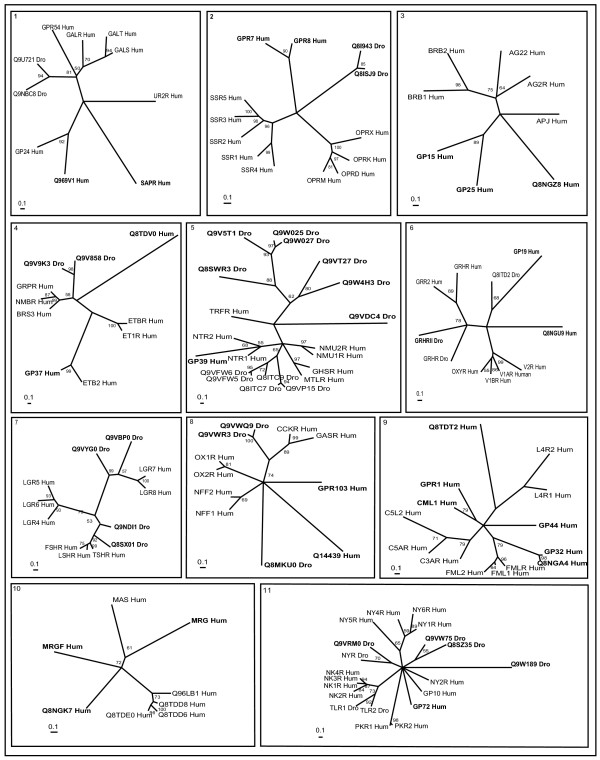
**Phylogenetic trees of peptide receptors (clusters 1–11)**. Trees were inferred as described in Methods (using TREE-PUZZLE 5.1 corrected using JTT substitution frequency matrix. Quartet-puzzling support percentage values from 10,000 puzzling steps are shown). Out-group not showed in the figure. The scale bars indicate a maximum likelihood branch length of 0.1 inferred substitutions per site. Orphan receptors are shown in bold letters. Cluster numbers are marked in the top left corner.

**Figure 2:**
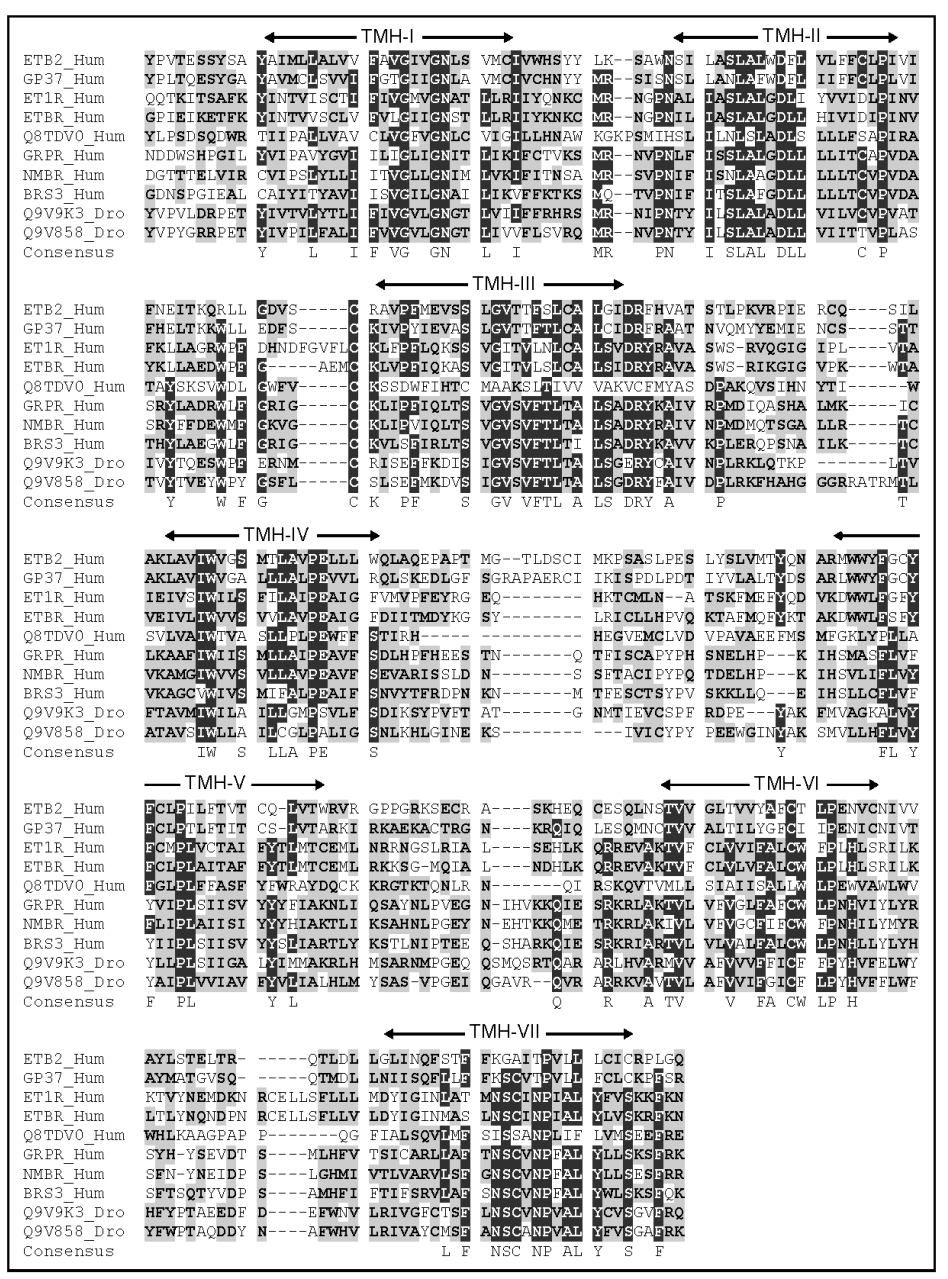
**Representative multiple sequence alignment of GPCR clusters**. GPCR sequences of ET1R_Hum, ETAR_Hum, ETBR_Hum, ETB2_Hum, GRPR_Hum, NMBR_Hum, BRS3_Hum, GP37_Hum, Q8TDV0_Hum, Q9V858_Dro and Q9V9K3_Dro belonging to cluster 4 were aligned with ClustalX. Sequence region comprising of TMH-1 to TMH-7 alone were considered for the analysis (Alignment was modified by deleting the extremely variable amino termini upstream of the first transmembrane helix and carboxyl termini downstream of the seventh transmembrane helix). Identical amino-acid residues in all aligned sequences are shaded in black and similar residues in gray and consensus residues are indicated below. Transmembrane helices (TMH) identified by the HMMTOP program are indicated.

**Figure 3:**
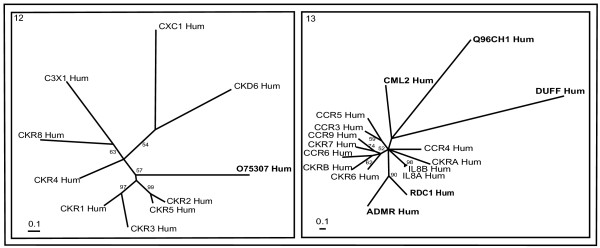
**Phylogenetic trees of chemokine receptors (clusters 12 and 13)**. The mode of deriving phylogenetic trees is as described in Methods and indications are as in Figure 2.

**Figure 4:**
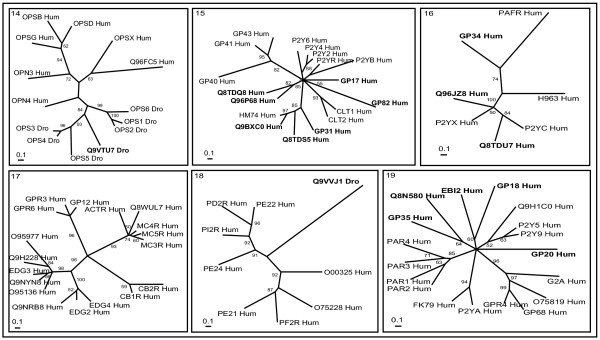
**Phylogenetic trees of nucleotide and lipid receptors (clusters 14–19)**. The mode of deriving phylogenetic trees is as described in Methods and indications are as in Figure 2.

**Figure 5:**
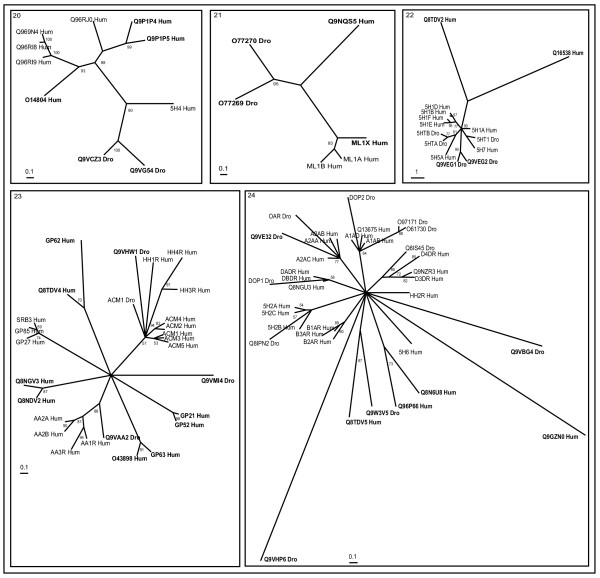
**Phylogenetic trees of biogenic amine receptors (clusters 20–24)**. The mode of deriving phylogenetic trees is as described in Methods and indications are as in the Figure 2 except for the cluster 22, where scale bar indicates a maximum likelihood branch length of 1.0 inferred substitutions per site.

**Figure 6:**
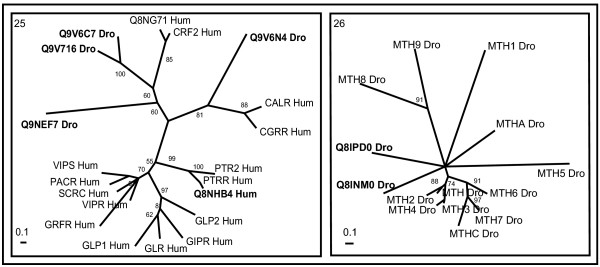
**Phylogenetic trees of class B (secretin) receptors (clusters 25 and 26)**. The mode of deriving phylogenetic trees is as described in Methods and indications are as in Figure 2.

**Figure 7:**
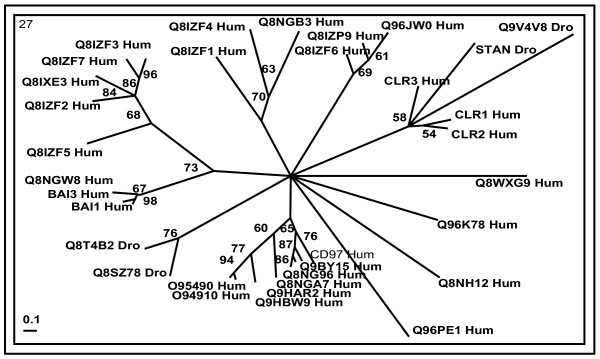
**Phylogenetic tree of cell adhesion receptors (cluster 27)**. The mode of deriving phylogenetic tree is as described in Methods and indications are as in Figure 2.

**Figure 8:**
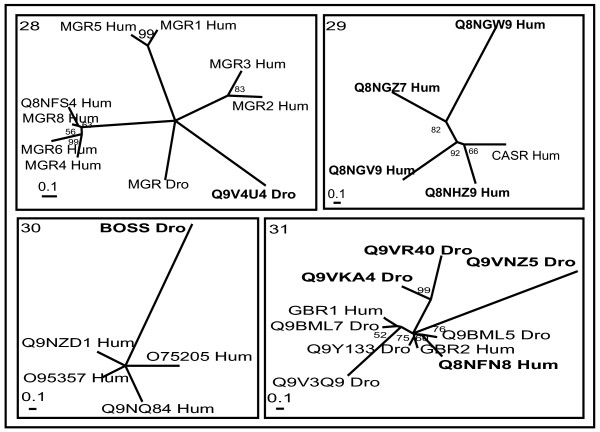
**Phylogenetic trees of class C (glutamate) receptors (clusters 28–31)**. The mode of deriving phylogenetic trees is as described in Methods and indications are as in Figure 2.

**Figure 9:**
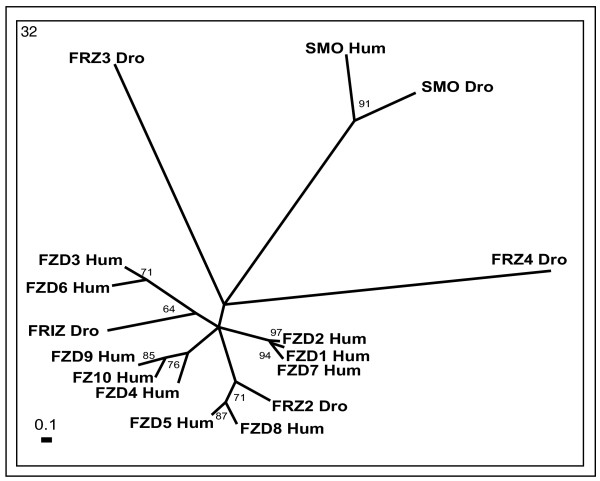
**Phylogenetic tree of frizzled/smoothened receptors (cluster 32)**. The mode of deriving phylogenetic tree is as described in Methods and indications are as in Figure 2.

Cross genome phylogenetic analysis of human and *Drosophila* non-olfactory receptors resulted in eight major groups. They are i) peptide receptors, ii) chemokine receptors, iii) nucleotide and lipid receptors iv) biogenic amine receptors v) secretin receptors vi) glutamate receptors vii) cell adhesion receptors and viii) frizzled receptors. These were further classified into 32 clusters (Table 1) with eleven clusters of peptide receptors, two clusters of chemokine receptors, six clusters of nucleotide and lipid receptors, five clusters of biogenic amine receptors, two clusters of secretin receptors, four clusters of glutamate receptors and one cluster each of cell adhesion and frizzled receptors (The combined phylogenetic and ligand analyses of human-*Drosophila* GPCRs are shown in Figures 1, 2, 3, 4, 5, 6, 7, 8, 9). About thirty one GPCR sequences could not be assigned to any of these clusters; these are discussed separately below as unassociated GPCRs. Our method sometimes resulted in clusters with members whose ligands belong to different chemical structure or classes and these results are discussed in detail below.

**Table 1:** List of GPCRs in each of the 32 clusters derived from phylogenetic analysis. Suffix _Hum and _Dro refers to human and *Drosophila* sequences respectively. Orphan receptors are shown in bold.

**CLUSTER 1**	**CLUSTER 2**	**CLUSTER 3**	**CLUSTER 4**	**CLUSTER 5**		**CLUSTER 6**	**CLUSTER 7**	**CLUSTER 8**
GALR_Hum	**GPR7_Hum**	AG22_Hum	BRS3_Hum	GHSR_Hum	Q9VFW6_Dro	**GP19_Hum**	FSHR_Hum	CCKR_Hum
GALS_Hum	**GPR8_Hum**	AG2R_Hum	ET1R_Hum	GP39_Hum	Q9VP15_Dro	GRHR_Hum	LGR4_Hum	GASR_Hum
GALT_Hum	OPRD_Hum	APJ_Hum	ETB2_Hum	MTLR_Hum	**Q9VT27_Dro**	GRR2_Hum	LGR5_Hum	NFF1_Hum
GP24_Hum	OPRK_Hum	BRB1_Hum	ETBR_Hum	NTR1_Hum	**Q9W025_Dro**	GRHR_Dro	LGR6_Hum	NFF2_Hum
Q969F8_Hum	OPRM_Hum	BRB2_Hum	**GP37_Hum**	NTR2_Hum	**Q9W027_Dro**	OXYR_Hum	LGR7_Hum	OX1R_Hum
**Q969V1_Hum**	OPRX_Hum	**GP15_Hum**	GRPR_Hum	NMU1R_Hum	Q9W4H3_Dro	**GRHRII_Dro**	LGR8_Hum	OX2R_Hum
Q9NBC8_Dro	**Q8I943_Dro**	**GP25_Hum**	NMBR_Hum	NMU2R_Hum	TRFR_Hum	Q8ITD2_Dro	LSHR_Hum	**Q14439_Hum**
Q9U721_Dro	**Q8ISJ9_Dro**	**Q8NGZ8_Hum**	**Q8TDV0_Hum**	Q8ITC7_Dro		**Q8NGU9_Hum**	**Q8SX01_Dro**	**GPR103_Hum**
**SAPR_Hum**	SSR1_Hum		**Q9V858_Dro**	Q8ITC9_Dro		V1AR_Hum	**Q9NDI1_Dro**	**Q8MKU0_Dro**
UR2R_Hum	SSR2_Hum		**Q9V9K3_Dro**	**Q8SWR3_Dro**		V1BR_Hum	**Q9VBP0_Dro**	**Q9VWQ9_Dro**
	SSR3_Hum			**Q9V5T1_Dro**		V2R_Hum	**Q9VYG0_Dro**	**Q9VWR3_Dro**
	SSR4_Hum			**Q9VDC4_Dro**			TSHR_Hum	
	SSR5_Hum			Q9VFW5_Dro				

**CLUSTER 9**	**CLUSTER 10**	**CLUSTER 11**	**CLUSTER 12**	**CLUSTER 13**	**CLUSTER 14**	**CLUSTER 15**	**CLUSTER 16**	**CLUSTER 17**

C3AR_Hum	MAS_Hum	GP10_Hum	C3X1_Hum	**ADMR_Hum**	OPN3_Hum	CLT1_Hum	**GP34_Hum**	ACTR_Hum
C5AR_Hum	**MRG_Hum**	**GP72_Hum**	CKD6_Hum	CCR3_Hum	OPN4_Hum	CLT2_Hum	H963_Hum	CB1R_Hum
C5L2_Hum	**MRGF_Hum**	NK1R_Hum	CKR1_Hum	CCR4_Hum	OPS1_Dro	**GP17_Hum**	P2YC_Hum	CB2R_Hum
**CML1_Hum**	**Q8NGK7_Hum**	NK2R_Hum	CKR2_Hum	CCR5_Hum	OPS2_Dro	**GP31_Hum**	P2YX_Hum	EDG2_Hum
FML1_Hum	Q8TDD6_Hum	NK3R_Hum	CKR3_Hum	CCR6_Hum	OPS3_Dro	GP40_Hum	PAFR_Hum	EDG3_Hum
FML2_Hum	Q8TDD8_Hum	NK4R_Hum	CKR4_Hum	CKR6_Hum	OPS4_Dro	GP41_Hum	**Q8TDU7_Hum**	EDG4_Hum
FMLR_Hum	Q8TDE0_Hum	NY1R_Hum	CKR5_Hum	CKR7_Hum	OPS5_Dro	GP43_Hum	**Q96JZ8_Hum**	GP12_Hum
**GP32_Hum**	Q96LB1_Hum	NY2R_Hum	CKR8_Hum	CKRA_Hum	OPS6_Dro	**GP82_Hum**		GPR3_Hum
**GP44_Hum**		NY4R_Hum	CXC1_Hum	CKRB_Hum	OPSB_Hum	HM74_Hum		GPR6_Hum
**GPR1_Hum**		NY5R_Hum	**O75307_Hum**	**CML2_Hum**	OPSD_Hum	P2Y2_Hum		MC3R_Hum
L4R1_Hum		NYR_Dro		**DUFF_Hum**	OPSG_Hum	P2Y4_Hum		MC4R_Hum
L4R2_Hum		PKR1_Hum		IL8A_Hum	OPSX_Hum	P2Y6_Hum		MC5R_Hum
**Q8NGA4_Hum**		PKR2_Hum		IL8B_Hum	Q96FC5_Hum	P2YB_Hum		O95136_Hum
**Q8TDT2_Hum**		**Q8SZ35_Dro**		CKR9_Hum	**Q9VTU7_Dro**	P2YR_Hum		O95977_Hum
		NY6R_Hum		**Q96CH1_Hum**		**Q8TDQ8_Hum**		Q8WUL7_Hum
		**Q9VRM0_Dro**		**RDC1_Hum**		**Q8TDS5_Hum**		Q9H228_Hum
		**Q9VW75_Dro**				**Q96P68_Hum**		Q9NRB8_Hum
		**Q9W189_Dro**				**Q9BXC0_Hum**		Q9NYN8_Hum
		TLR1_Dro						
		TLR2_Dro						

**CLUSTER 18**	**CLUSTER 19**	**CLUSTER 20**	**CLUSTER 21**	**CLUSTER 22**	**CLUSTER 23**		**CLUSTER 24**	

O00325_Hum	**EBI2_Hum**	5H4_Hum	ML1A_Hum	5H1A_Hum	AA1R_Hum	**GP63_Hum**	5H2A_Hum	HH2R_Hum
O75228_Hum	FK79_Hum	**O14804_Hum**	ML1B_Hum	5H1B_Hum	AA2A_Hum	GP85_Hum	5H2B_Hum	O61730_Dro
PD2R_Hum	**GP18_Hum**	Q969N4_Hum	**ML1X_Hum**	5H1D_Hum	AA2B_Hum	HH1R_Hum	5H2C_Hum	O97171_Dro
PE21_Hum	**GP20_Hum**	Q96RI8_Hum	**O77269_Dro**	5H1E_Hum	AA3R_Hum	HH3R_Hum	5H6_Hum	OAR_Dro
PE22_Hum	**GP35_Hum**	Q96RI9_Hum	**O77270_Dro**	5H1F_Hum	ACM1_Dro	HH4R_Hum	A1AB_Hum	Q13675_Hum
PE24_Hum	GP68_Hum	Q96RJ0_Hum	**Q9NQS5_Hum**	5H5A_Hum	ACM1_Hum	**O43898_Hum**	A1AD_Hum	Q8IPN2_Dro
PF2R_Hum	GPR4_Hum	**Q9P1P4_Hum**		5H7_Hum	ACM2_Hum	**Q8NDV2_Hum**	A2AA_Hum	Q8IS45_Dro
PI2R_Hum	O75819_Hum	**Q9P1P5_Hum**		5HT1_Dro	ACM3_Hum	**Q8TDV4_Hum**	A2AB_Hum	**Q8N6U8_Hum**
**Q9VVJ1_Dro**	P2Y5_Hum	**Q9VCZ3_Dro**		5HTA_Dro	ACM4_Hum	**Q9VAA2_Dro**	A2AC_Hum	Q8NGU3_Hum
	P2Y9_Hum	**Q9VG54_Dro**		5HTB_Dro	ACM5_Hum	**Q9VHW1_Dro**	B1AR_Hum	**Q8TDV5_Hum**
	P2YA_Hum			**Q16538_Hum**	**GP21_Hum**	**Q9VMI4_Dro**	B2AR_Hum	**Q96P66_Hum**
	PAR1_Hum			**Q8TDV2_Hum**	GP27_Hum	SRB3_Hum	B3AR_Hum	**Q9GZN0_Hum**
	PAR2_Hum			**Q9VEG1_Dro**	**GP52_Hum**		D3DR_Hum	Q9NZR3_Hum
	PAR3_Hum			**Q9VEG2_Dro**	**GP62_Hum**		D4DR_Hum	**Q9VBG4_Dro**
	PAR4_Hum						DADR_Hum	**Q9VE32_Dro**
	**Q8N580_Hum**						DBDR_Hum	**Q9VHP6_Dro**
	Q9H1C0_Hum						DOP1_Dro	**Q9W3V5_Dro**
	Q9UNW8_Hum						DOP2_Dro	

**CLUSTER 25**	**CLUSTER 26**	**CLUSTER 27**		**CLUSTER 28**	**CLUSTER 29**	**CLUSTER 30**	**CLUSTER 31**	**CLUSTER 32**

CALR_Hum	MTH_Dro	**BAI1_Hum**	**Q8NG96_Hum**	MGR_Dro	CASR_Hum	O75205_Hum	GBR1_Hum	FRIZ_Dro
CGRR_Hum	MTH1_Dro	**BAI3_Hum**	**Q8NGA7_Hum**	MGR1_Hum	**Q8NGV9_Hum**	O95357_Hum	GBR2_Hum	FRZ2_Dro
CRF2_Hum	MTH2_Dro	CD97_Hum	**Q8NGB3_Hum**	MGR2_Hum	**Q8NGW9_Hum**	Q9NQ84_Hum	**Q8NFN8_Hum**	FRZ3_Dro
GIPR_Hum	MTH3_Dro	**CLR1_Hum**	**Q8NGW8_Hum**	MGR3_Hum	**Q8NGZ7_Hum**	Q9NZD1_Hum	Q9BML5_Dro	FRZ4_Dro
GLP1_Hum	MTH4_Dro	**CLR2_Hum**	**Q8NH12_Hum**	MGR4_Hum	**Q8NHZ9_Hum**	**BOSS_Dro**	Q9BML7_Dro	FZ10_Hum
GLP2_Hum	MTH5_Dro	**CLR3_Hum**	**Q8SZ78_Dro**	MGR5_Hum			Q9V3Q9_Dro	FZD1_Hum
GLR_Hum	MTH6_Dro	**O94910_Hum**	**Q8T4B2_Dro**	MGR6_Hum			**Q9VKA4_Dro**	FZD2_Hum
GRFR_Hum	MTH7_Dro	**O95490_Hum**	**Q8WXG9_Hum**	MGR8_Hum			**Q9VNZ5_Dro**	FZD3_Hum
PACR_Hum	MTH8_Dro	**Q8IXE3_Hum**	**Q96JW0_Hum**	Q8NFS4_Hum			**Q9VR40_Dro**	FZD4_Hum
PTR2_Hum	MTH9_Dro	**Q8IZF1_Hum**	**Q96K78_Hum**	**Q9V4U4_Dro**			Q9Y133_Dro	FZD5_Hum
PTRR_Hum	MTHA_Dro	**Q8IZF2_Hum**	**Q96PE1_Hum**					FZD6_Hum
Q8NG71_Hum	MTHC_Dro	**Q8IZF3_Hum**	**Q9BY15_Hum**					FZD7_Hum
**Q8NHB4_Hum**	**Q8INM0_Dro**	**Q8IZF4_Hum**	**Q9HAR2_Hum**					FZD8_Hum
**Q9V6C7_Dro**	**Q8IPD0_Dro**	**Q8IZF5_Hum**	**Q9HBW9_Hum**					FZD9_Hum
**Q9V6N4_Dro**		**Q8IZF6_Hum**	**Q9V4V8_Dro**					**SMO_Dro**
**Q9V716_Dro**		**Q8IZF7_Hum**	**STAN_Dro**					**SMO_Hum**
SCRC_Hum		**Q8IZP9_Hum**						
VIPR_Hum								
VIPS_Hum								

### Peptide receptors

Clusters 1 to 11 comprise of peptide receptors (Figure 1). The size of peptide ligands can vary from two amino acids to as many as 50. Some of the natural peptide ligands include apelin, bombesin, calcitonin, endothelin, galanin, gastrin, ghrelin, neurotensin, neuropeptide B, W, Y, orexin, oxytocin, relaxin, somatostatin, urocortins, etc. These receptors are involved in many human diseases including chronic inflammatory diseases, degenerative diseases, autoimmune diseases, cancer, cardiovascular diseases etc, thus they could be of new therapeutic targets [24,25].

Receptors with known ligands in cluster 1 binds to galanins or kisspeptins or cyclic peptides. *Drosophila* allostatin receptors (DARs) (Q9NBC8_Dro and Q9U721_Dro) are very closely related to galanin receptors [26]. Receptors, Q969V1_Hum and Q96S47_Hum, are closely related to GP24_Hum receptor that bind to melanin-concentrating hormone and may have similar cyclic peptides as their ligands. As the name suggests, orphan receptor, SAPR_Hum, does not bind to somatostatins and angiotensins [27] since it is distantly related to GP24_Hum and UR2R_Hum receptors in this tree. Instead, this receptor may bind to similar cyclic peptides.

Cluster 2 consists of receptors for opioid, somatostatin and neuropeptide (NPB or NPW) ligands forming different branches. Opioids and somatostatins are obtained from preprocessing of larger precursor peptides. It is known that GPR7_Hum and GPR8_Hum bind to NPB/W ligands [28]. *Drosophila* orphan receptors, Q8ISJ9_DRo and Q8I943_Dro branch is close to somatostatin receptors and might bind to ligands similar to somatostatins. Small peptide (apelin, angiotensin, and bradykinin) receptors comprise of cluster 3. The human orphan receptors encoded by GPR15_Hum, GPR25_Hum and Q8NGZ8_Hum are related to APJ_Hum and show significant amino acid identity suggesting these might bind to small peptide endogenous ligands.

Cluster 4 comprises of endothelin and bombesin receptors with known ligands (ET1R_Hum, ETAR_Hum and ETBR_Hum, gastrin-releasing peptide receptor (GRPR_Hum), the neuromedin B receptor (NMBR_Hum) and bombesin receptor (BRS3)). *Drosophila* orphan receptors, Q9V9K3_Dro and Q9V858_Dro, share the branch with bombesin, GRPR and NMBR receptors. They share many conserved amino acids, known to be important for high affinity binding of gastrin-releasing peptide (GRP) and bombesin to GRPR and NMB binding to NMB-R [29-31] (Figure 2). This suggests Q9V9K3_Dro and Q9V858_Dro might bind to similar neuropeptide(s) for its activation. Human orphan receptor GPR37_Hum is closely related to ETB2_Hum suggesting it may bind to endothelin-like peptides. Q8TDV0_Hum is sequentially similar to both galanin (cluster 1) and bombesin receptors but sub-clustering of peptide receptors by maximum likelihood method has placed it in this cluster suggesting closer association of these two clusters.

Cluster 5 is composed of receptors for neurotensin (NT), neuromedin U (NMU), motilin, growth hormone secretagogue, thyrotropin-releasing hormone and some of PRX-amide peptides. GPR39_Hum is closely related to NT receptors and might bind to neurotensin ligands. *Drosophila* receptors, Q8ITC7_Dro, Q9VFW5_Dro, Q9VFW6_Dro, Q8ITC9_Dro and Q9VP15_Dro form a separate branch, which are closely related to vertebrate neuromedin receptors and they bind to PRXa pyrokinins or FXPRXamide or Cap2b-like peptides (FPRXamide) or ecdysis triggering hormones (PRXamide) (Park et al. 2002). Q9VDC4_Dro forms a distinct branch and is sequentially close to GHSR_Hum, TRFR_Hum, Q8ITC7_Dro and Q9VFW5_Dro and might bind to neuropeptides. *Drosophila* orphan receptors, Q9W4H3_Dro, Q9VT27_Dro, Q8SWR3_Dro, Q9V5T1_Dro, Q9W025_Dro and Q9W027_Dro, branch out from that of TRFR_Hum and might form a separate family of receptors binding to novel neuropeptide ligands. Supporting our analysis, Q9W025_Dro and Q9W027_DRo were reported as first receptors specific for *Drosophila* myosuppressins (Drome-MS) [32] and Q9W4H3_Dro was reported as neuropeptide proctolin binding receptor [33]. Q9VT27_Dro is very closely related to Q9W4H3_Dro and might bind to proctolin or similar neuropeptide ligands for its activation.

Cluster 6 consists of peptide hormone receptors binding arginine vasopressin (AVP) or growth hormone releasing hormone or oxytocin or gonadotropin-releasing hormone II or crustacean cardioactive peptide (CCAP) or corazonin or adipokinetic hormone (AKH) (Park et al. 2002). GP19_Hum is related to *Drosophila* CCAP receptor (Q8ITD2_Dro) that is activated by CCAP and AKH, but not by AVP. Thus, CCAP and AKH might as well bind to GP19_Hum for its activation. *Drosophila* gonadotropin-releasing hormone and/or corazonin receptor (GRHR_Dro) and putative corazonin (GRHR II) receptor clusters well with human counterparts (GRHR_Hum and GRR2_Hum) suggesting early evolution of GRHR receptors. Q8NGU9_Hum forms a separate branch, but shares sequence similarity with AVP receptors and might bind to similar neuropeptide ligands.

Cluster 7 comprises leucine-rich repeat-containing G protein-coupled receptors (LGR) like glycoprotein receptors, follicle stimulating hormone receptor (FSHR_Hum), thyroid-stimulating hormone receptor (TSHR_Hum), luteinizing hormone receptor (LSHR_Hum) and receptors binding to relaxin. These are unique in having a large N-terminal extracellular (ecto) domain containing leucine-rich repeats important for interaction with the glycoprotein ligands and are classified into three sub-groups [34]. Our analysis also shows that there are three LGR subfamilies: (i) the glycoprotein hormone receptors LSHR_Hum, FSHR_Hum, TSHR_Hum, Q8SX01_Dro and Q9NDI1_Dro (ii) LGR4_Hum LGR5_Hum and LGR6_Hum (iii) LGR5_Hum, LGR7_Hum and LGR8_Hum, Q9VBP0_Dro, and Q9VYG0_Dro. *Drosophila* orphan receptors Q8SX01_Dro and Q9NDI1_Dro are closely related to human glycoprotein hormone receptors and might bind to glycoprotein hormones. Q9VBP0_Dro and Q9VYG0_Dro are very similar in their overall domain architecture to LGRs with long N-termini, but their similar relationship in extracellular domain arrangements are also evident from this phylogenetic analysis without considering the N and C termini.

Cluster 8 consists of peptide receptors with known ligands such as gastrin (GAS), cholecystokinin (CCK), orexin (OXR) and neuropeptide FF (NFF) or morphine modulating peptides. GPR103_Hum (Q96P65) is closely related to neuropeptide FF receptors, as predicted by our phylogenetic analysis and previous prediction on human GPCRs [12]. Subsequently, GPR103 was characterized and a novel RF-amide peptide, P52 was shown to be its ligand [35]. *Drosophila* orphan receptors, Q9VWR3_Dro (CCKLR-17D1) and Q9VWQ9_Dro (CCKLR-17D3), are related to each other and branch off from the cholecystokinin (CCK) receptors and might have cholecystokinin as its natural ligand. Q14439_Hum branch off orexin receptors that bind to two novel neuropeptides, orexin-A and B, derived from a common prepro-orexin precursor by proteolytic processing [36].

The receptors with known ligands binding to chemotactic substances (hydrophilic peptides, N-formyl-methionyls (FML) and anaphylactic complement factors) are part of cluster 9. These ligands are structurally very diverse but functionally related peptides. Human orphan receptors, GP32_Hum and Q8NGA4_Hum branch out early from FML receptors and may probably bind to smaller hydrophilic peptides. L4R1_Hum, L4R2_Hum and Q8TDT2_Hum form a separate branch distant from other chemotactic peptide receptors with out bootstrap support. CML1_Hum and GPR1_Hum form a separate branch distinct from the other branches, and also GPR44_Hum forming an individual branch. Prediction of ligands for these receptors is not possible using this phylogenetic tree, but these receptors may be activated by chemotactic substances [37].

Mas proto-oncogene, Mas-related genes (MRGs) and sensory neuron-specific G protein-coupled receptors (SNSRs) form cluster 10. Angiotensin (1–7) has been identified as an endogenous ligand for the G protein-coupled receptor Mas [38]. SNSRs are activated by proenkephalin A peptide fragments, like bovine adrenal medulla peptide 22 (BAM22). Some MRGs and SNSRs are expressed in nociceptive sensory neurons suggesting that they could be involved in pain sensation or its modulation. Previous studies also suggest that ligands for MRG receptors may include neuropeptides that modulate pain sensitivity [39]. Human orphan receptor Q8NGK7_Hum is closely related to MRG receptor.

All receptors with known ligands in cluster 11 are neuropeptide receptors. *Drosophila* tachykinin-like peptide receptors (TLR1_Dro and TLR2_Dro) and human neurokinin receptors (NK1-4R_Hum) form a closely-knit branch. PKR1_Hum (Q8NFJ7) and PKR2_Hum (Q8NFJ6) form a separate branch of receptors that bind to prokineticins [40]. Q9VRM0_Dro is closely related to *Drosophila* receptor NYR_Dro that bind to neuropeptide Y. Q9VRM0_Dro might probably bind to similar neuropeptides. Neuropeptide Y binding receptors (NY1R_Hum, NY4R_Hum, NY5R_Hum and NY6R_Hum (Q99463)) form a separate branch. The human prolactin-releasing peptide (PrRP) binding GPR10_Hum forms a separate branch in this phylogenetic tree [41]. *Drosophila* orphan receptors, Q9VW75_Dro and Q8SZ35_Dro constitute a separate branch close to other neuropeptide receptors that might functionally be activated by neuropeptides. Similarly, orphan receptor GP72_Hum forms a new branch. *Drosophila* orphan receptor Q9W189_Dro is a very distantly related member and was only grouped into this cluster by blastp results.

### Chemokine receptors

Chemokine receptors are phylogenetically represented by two clusters 12 and 13 (Figure 3). Chemokines are important molecules in inflammatory responses, as immunomodulators and they also have critical functions in lymphopoiesis [42]. There are no *Drosophila* members belong to this group of receptors suggesting these receptors might be recent in evolutionary origin. They have been divided into two subfamilies on the basis of the arrangement of the two disulphide-bond forming N-terminal cysteine residues, CXC and CC. Many human CXC chemokines that mainly act on neutrophils are clustered at chromosome 4q12–13, while many CC chemokines that mainly act on monocytes are located in another cluster at chromosome 17q11.2. Our phylogenetic analysis has also divided chemokine receptors into two major clusters, concurrent with that of chemokine classes, suggesting co-evolution of receptors and ligands [43].

Cluster 12 consists of receptors associated with CC type chemokines. As reported previously through earlier approach [12] O75307_Hum (CRAM-A) might bind to CC-type chemokine ligand. Cluster 13 consists of both CXC and CC-type receptors. ADMR_Hum and Q8NE10_Hum (RDC1) form a branch whereas Duff antigen and Q96CH1_Hum are distantly related to CML2_Hum. These two branches are associated to chemokine receptors based on BLASTP similarity at an E-value significance of 5e-04 and 7e-07, respectively, with other members of this cluster.

### Nucleotide and lipid receptors

Nucleotide and lipid receptors consists of six clusters (Figure 4), except for cluster 14 (opsins) and cluster 18 (receptors binding ligands are derivatives of arachidonic acid) there are no counter parts from *Drosophila*. Opsins are included in cluster 14 that are activated by isoprenoid ligands. *Drosophila* opsins show significantly high homology to human opsins. There is strong conservation of the retinal binding site and other regions suggesting that they are derived from a common ancestor and diverged thereafter retaining structural and functional features [44]. *Drosophila* receptor Q9VTU7_Dro is closely related to OPS3–5_Dro receptors, which are localized in the inner-cells of the *Drosophila* eye (either R7 or R8 cells). This suggests Q9VTU7_Dro might be localized in the inner cells of *Drosophila* eye.

Receptors for pyramidine or purine nucleotides, cysteinyl leukotriene, nicotinic acid (niacin; pellagra preventing factor) and short, medium and long chain fatty acids make up cluster 15. Q9BXC0_Hum (GPR81), Q8TDS5_Hum and GP31_Hum share the branch with closely related nicotinic acid (HM74_Hum) receptor [45] and might have similar carboxylic acids as their ligands. Q8TDQ8_Hum and Q96P68_Hum are related to each other as well as to P2Y receptors and may bind to P2Y nucleotides. GP17_Hum and GP82_Hum receptors are distantly related to other members in this cluster and might represent potential new subfamilies binding to nucleotide or lipids.

Cluster 16 is a heterogeneous group of receptors binding to lipids, nucleotides, modified nucleotides and platelet activating factor (PAF). Orphan receptor Q8TDU7_Hum (GPR86) is closely related to platelet ADP-binding receptor (P2YC_Hum). Q96JZ8_Hum (GPR87) is closely related to UDP-glucose receptor (P2YX_Hum) and might bind to a modified nucleotide ligand. GPR34_Hum forms a separate branch which is distantly related to PAFR_Hum. No prediction of ligands is possible for GPR34_Hum with this phylogenetic tree.

Cluster 17 consists of lipid receptors (cannabinoids, lysophospholipid sphingosine 1-phosphate (S1P)) and exceptionally some of the peptide receptors (melanocortin peptides derived from processing of pro-opiomelanocortin) are represented in different branches. Although they bind to different ligands, they identify each other during sequence searches and display 23–29% sequence identity. The functionally important motifs are fairly conserved [46] (please see Additional data file 2). Indeed, this unusual branching including peptide and lipid receptors has been noted earlier by Methner's and Fredicksson's groups [12,16].

Cluster 18 is composed of receptors binding to prostaglandins, prostacyclins and thromboxanes. All these ligands are derivatives of arachidonic acid (AA), which serves as the precursor via the cyclooxygenase (COX) pathway. *Drosophila* orphan receptor Q9VVJ1_Dro within this tree might bind to ligands derived from AA by the action of COX.

Cluster 19 is also a heterogeneous group of receptors consisting of protease-activated receptors, psychosine receptors, lysophosphatidylcholine and sphingosylphosphorylcholine. Ovarian cancer G-protein-coupled receptor 1 (OGR1), previously described as a receptor for sphingosylphosphorylcholine, acts as a proton-sensing receptor stimulating inositol phosphate formation [47], whereas GPR4 is also involved in pH homeostasis, but elicits cyclic AMP formation [48]. OGR1 (GPR68) and GPR4 are different from other sphingosylphosphorylcholine binding endothelial differentiation gene (EDG) receptors. Orphan P2Y receptors in this cluster are misnomers since they do not cluster with the classical neuropeptide receptors (cluster 15 and 16) and instead appear to be co-clustered with members of this heterogeneous cluster. Either they may have uncommon nucleotide(s) as natural ligand or despite their structural similarity to the P2Y family they may not be nucleotide receptors [49]. GP35_Hum and Q8N580_Hum, EBI2_Hum and GP18_Hum and GP20_Hum cluster as separate branches and are distantly related to members of other branches but probably bind to lipids as their natural ligands.

### Biogenic amine receptors

Biogenic amine receptors consists of five clusters mainly consisting of trace amine; melatonin; serotonin receptors; histamines, muscarinic acetylcholine, adenosine and histamine; dopamine, octopamine and adrenaline receptors (Figure 5). In these clusters fairly good intermixing of human and *Drosophila* receptors is observed. This suggests biogenic amine receptors have ancient evolutionary origin as they are observed in invertebrates to higher vertebrates. Cluster 20 is represented mainly by trace amine (TA) receptors (Figure 5). Trace amines binding these receptors are believed to play an important role in human disorders such as depression, attention deficit disorder, schizophrenia and parkinson's disease [50]. They form a subfamily of GPCRs, distinct from, but related to serotonin (5-HT), Norepinephrine (NE) and dopamine (DA) receptors. *Drosophila* orphan receptors Q9VG54_Dro and Q9VCZ3_Dro are closely related to 5H4_Hum. Q9P1P4_Hum (GPR57) and Q9P1P5_Hum (GPR58) are closely related to Q96RJ0_Hum (TA1). Similarly O14804_Hum, a putative neurotransmitter receptor (PNR) is closely related to trace amine (Q969N4_Hum, Q96RI8_Hum, and Q96RI9_Hum) receptors.

Cluster 21 consists of melatonin receptors (ML1A_Hum, ML1B_Hum and ML1X_Hum) and other related orphan receptors (O77269_Dro, O77270_Dro, and Q9NQS5_Hum). Melatonin receptors bind to and are activated by biogenic amine 5-methoxy-N-acetyltryptamine (melatonin). The melatonin-related receptor (ML1X_Hum), despite sharing considerable amino acid sequence identity with other melatonin receptors, does not bind melatonin [51]. The receptors in this cluster show considerable sequence similarity to neuropeptide Y (NPY) receptors than other biogenic amine receptors and were previously grouped along with NPY receptors [12].

All receptors with known ligands of Cluster 22 consist of serotonin receptors. These are structurally distinct from serotonin receptors in cluster 24. *Drosophila* orphan receptors Q9VEG1_Dro and Q9VEG2_Dro form a separate branch but are closely related to other serotonin receptors in this tree and might have similar ligand (s) for its activation. Q8TDV2_Hum and Q16538_Hum (Protein A-2), however, are distantly related to other receptors in this tree and were placed only based on BLASTP similarity.

Receptors of biogenic amines (muscarinic acetylcholine, adenosine and histamine) and many orphan receptors are all placed in different branches in cluster 23. *Drosophila* orphan receptor Q9VHW1_Dro branch out along with muscarinic acetylcholine and histamine receptors in this tree and might bind to acetylcholine or histamines for its activation. Q9VAA2_Dro is closely related to that of adenosine receptors. Super conserved receptors expressed in brain (SRB1-3) from vertebrate species form a separate branch and might represent potential novel subfamily of GPCRs binding to undiscovered endogenous biogenic amine ligands [52]. High-affinity lysophosphatidic acid (LPA) receptor homologs O43898_Hum and GPR63_Hum form a distinct branch. Similarly, orphan receptors GP21_Hum and GP51_Hum, GPR62_Hum and Q8TDV4_Hum, Q8NDV2_Hum (GPR26) and Q8NGV3_Hum and Q9VMI4_Dro form a distinct branch, suggesting only distant relationship with other members of the cluster.

Receptors of biogenic amines (dopamine, histamine, octopamine and adrenaline), few serotonergic receptors and many orphan receptors are represented in different branches in cluster 24. *Drosophila* dopamine 2-like receptor (DD2R), Q8IS45_Dro, groups well with the human counterparts suggesting that their evolution extends much before *Drosophila*. Interestingly, DOP2_Dro is grouped with the adrenaline receptors instead with dopaminergic receptors and shows similar sequence identity (40–48%) with vertebrate alpha 1-, and beta-adrenergic, and D1-like, D2-like dopaminergic and serotonergic receptors. This *Drosophila* receptor has been discussed as a novel structural class of dopamine receptors [53]. *Drosophila* octopamine receptor isoforms in mushroom bodies (OAMB) (O97171_Dro and O61730_Dro) branch out with human alpha 1 adrenergic (A1A (A, B and D) _Hum) receptors since they share high sequence identity (52–55%) in TM regions with alpha 1 adrenergic receptors [54]. Q9VE32_Dro branches out from human alpha 2 adrenergic receptors and may have adrenaline as its ligand for activation. Orphan striatum-specific G protein-coupled receptor (STRG or Q9GZN0_Hum), though grouped with biogenic amine receptors, may represent a novel subtype of GPCR due to the lack of conservation of key functional residues [55]. Orphan receptors, Q9W3V5_Dro and Q8TDV5_Hum, Q96P66_Hum and Q8N6U8_Hum, Q9VHP6_Dro and Q9VBG4_Dro form their own branch sharing distant relationship with other receptors in this tree and might represent potential novel subfamilies of biogenic amine GPCRs.

### Class B (secretin) receptors

Class B receptors are represented by two clusters (25 and 26) consisting of classical hormone receptors and *Drosophila* methuselah (MTH) like proteins (Figure 6). The ligands for receptors of cluster 25 are structurally related polypeptide hormones of 27–141 amino-acid residues (pituitary adenylate cyclase-activating polypeptide (PACAP), secretin, calcitonin, corticotropin-releasing factor (CRF), urocortins, growth-hormone-releasing hormone (GHRH), vasoactive intestinal peptide (VIP), glucagon, glucagon-like peptides (GLP-1, GLP-2) and glucose-dependent insulinotropic polypeptide (GIP). *Drosophila* orphan receptors, Q9V716_Dro and Q9V6C7_Dro are closely related to the human receptor for Corticotropin releasing factor receptor (CRF) which binds to urocortins. Q9V6N4_Dro, Q9VYH9_Dro and Q9NEF7_Dro are related to calcitonin (CALR_Hum) and calcitonin gene-related peptide type 1 receptors (CGRR_Hum). Three small accessory proteins, called receptor activity-modifying proteins (RAMPs), interact with these calcitonin receptors and can generate six pharmacologically distinct receptors. If this phenomenon of RAMP-enabled receptor diversity exists in other receptors, then it will further complicate the ligand-receptor interactions of GPCRs, assuming they still bind to structurally similar ligands. Human orphan receptor, Q8NHB4_Hum, is very closely related to PTRR_Hum receptor binding to parathyroid hormone and parathyroid hormone-related protein (PTHrP). Methuselah receptors and its paralogs of *Drosophila* solely represent cluster 26. The *Drosophila* mutant methuselah (MTH) was identified from a screen for single gene mutations that extended average lifespan of an organism and also increased resistance to several forms of stress, including starvation, heat, and oxidative damage [56]. There are no obvious homologues of these receptors within human or *C. elegans *genomes. *Drosophila* receptors, Q8INM0_Dro, Q8IPD0_Dro and Q95NU7_Dro, are closely related to previously identified MTH members and may be new paralogs of these receptors.

### Cell adhesion receptors

Large number of GPCRs with long extracellular N-termini, containing GPCR proteolytic site (GPS) domain, are represented in cluster 27 (Figure 7). Several of these receptors also have one or many functional domains such as epidermal growth factor (EGF), leucine rich repeat (LRR), hormone-binding domain (HBD) and immunoglobulin (Ig) domains [16]. These form several distantly related branches. Except CD97_Hum, all the receptors in this cluster are orphans with no known ligands [57]. There are only four *Drosophila* sequences representing these receptors.

### Class C (glutamate) receptors

Receptors of Class C are divided mainly into four clusters (28–31): metabotropic glutamate receptors (MGR), γ-aminobutryic acid (GABA) receptors, calcium-sensing receptors (CASR) and retinoic acid-inducible G-protein-coupled receptors (RAIG) (Figure 8).

Cluster 28 consists of human and *Drosophila* MGRs. Human MGRs are sub-grouped into three different branches: first contains MGR1_Hum and MGR5_Hum and second contains MGR2_Hum and MGR3_Hum. The third branch, including MGR4_Hum, 6–8 and *Drosophila* MGRs represent a separate subgroup [58]. *Drosophila* orphan receptor Q9V4U4_Dro is closely related to MGR_Dro and might bind to glutamate for its activation.

Calcium-sensing receptor (CASR_Hum) forms cluster 29 along with a set of orphan receptors (Q8NHZ9_Hum, Q8NGV9_Hum, Q8NGW9_Hum and Q8NGZ7_Hum). These orphan receptors either may have ligands and/or function similar to that of CASR_Hum or they may act as pheromone/olfactory receptors. Phylogenetic tree of most members (including olfactory, putative pheromone, and sweet and amino acid taste receptors) of family 3 GPCRs across different genomes (Catfish (*Ictalurus punctatus*), *Caenorhabditis elegans*, *Drosophila melanogaster*, Japanese pufferfish (*Fugu rubripes*), Goldfish (*Carassius auratus*), human (*Homo sapiens sapiens*), mouse (*Mus musculus*), rat (*Rattus norvegicus*) and Salmon (*Oncorhynchus masou*)) have shown CASR_Hum forms a separate branch part of pheromone/olfactory cluster of class C GPCRs [59]. To note that olfactory and gustatory/taste receptors are not considered in this work.

Cluster 30 consists of retinoic acid-inducible G-protein-coupled receptors (RAIG). RAIGs have short (30–50 amino acids) extracellular amino-terminal domains (ATDs) as opposed to the other receptors currently assigned to class C. BOSS_Dro also has short ATD and branch out very early with the members of RAIGs and may represent new single member subfamily of class C receptors.

The GABA_B _receptors are present in cluster 31. It is represented by four sub-branches, of which three are GABA_B_R1-3_Hum type receptors and fourth sub-branch of *Drosophila* orphan receptors (Q9VKA4 and Q9VR40) related to that of GABA receptors. GABA_B3 _is exclusively present in *Drosophila* as separate branch whose function is not yet known. Previous results have only been able to functionally characterize D-GABA_B_R1 and R2 when the two subtypes are co-expressed either in *Xenopus laevis *oocytes or mammalian cell lines, whilst D-GABA_B_R3 was inactive in any combination. This suggests D-GABA_B_R3 requires a counterpart other than D-GABA_B_R1 and R2 to form a functional heterodimer [60]. Thus the current clustering approach suggests that Q9VKA4_Dro or Q9VR40_Dro may interact with D-GABA_B_R3 and form a functional heterodimer.

### Frizzled/smoothened receptors

Cluster 32 comprises receptors with a long (about 200-amino acid) N-terminus and conserved cysteine rich domains (CRD) which are likely to participate in Wnt ligand binding (Figure 9). These receptors control the specification of cell fate, cell adhesion, migration, polarity and proliferation [61]. This cluster is represented by ten human (FZD1-10) and four *Drosophila* (FRZ1-4) frizzled receptors together with smoothened (SMO_Hum and SMO_Dro) receptors. The topology of the phylogenetic tree shows one smoothened and four frizzled branches. FRZ1_Dro is closely related to human FZD3_Hum and FZD6_Hum. FRZ2_Dro is related to FZD5_Hum and FZD8_Hum, whereas FRZ3_Hum and FRZ4_Hum form separate branches distantly related to other receptors.

### Unassociated GPCRs

Thirty one GPCR sequences could not be included in any cluster with appreciable bootstrap values or BLASTP similarity. This can either be viewed as members of single member clusters with certain atypical parts of their sequences that could be a result of chimeric origin of the receptors or due to evolutionary pressure not shared by their closest phylogenetic neighbors [62]. We have therefore placed these receptors separately as unassociated GPCRs, although these receptors clearly do not belong to the same group (see Additional data file 1). Most of the unassociated receptors remain as orphan receptors.

## Conclusion

The phylogenetic analyses performed using human and *Drosophila* GPCRs suggest that the sequences can be divided into 32 clusters and reveals unexpected level of similarity between human and *Drosophila* GPCRs. 21 clusters group *Drosophila* and human GPCRs together suggesting high evolutionary conservation across species for GPCR sequences. There are 10 clusters, four of nucleotide-lipid receptors three clusters of peptide receptors and two clusters of chemokine and one cluster of glutamate receptors that do not contain any representation from *Drosophila* GPCRs in our current dataset of sequences considered. Perhaps the immune-related receptors, such as the chemokine ones, are not either recognized yet or not present in lower organisms such as *Drosophila*. If there is a clear absence of such classes of receptors, this might also suggest that immune defense is regulated by proteins other than GPCRs in *Drosophila*. Interestingly, there is one cluster of secretin *Drosophila* receptors where there is no human representation. These proteins are involved in aging in *Drosophila*. Furthermore, in this analysis, we also notice that out of the 21 clusters that co-cluster human and *Drosophila* GPCRs, *Drosophila* GPCRs remain isolated sub-clusters in 12 of them leaving behind only nine clusters that allow easy inter-mixing of the two sets of sequences. This includes 3 clusters each of peptide and biogenic amine receptors and one cluster each of class B, C and frizzled receptors.

The current clustering analysis provides ligand class association to 52 *Drosophila* (Table 2) and 95 human orphan receptors could be associated with probable ligand classes using co-clustering principles as earlier observed within human GPCR sequences alone [12]. Further, similar cellular localizations have been suggested for *Drosophila* orphan receptors that belong to the opsin family (cluster 14). GPCRs with similar extracellular domain architecture also co-cluster suggesting this similarity is encoded even within the GPCR domain. Further this analysis also suggests dimerizing partner (Q9VKA4_Dro or Q9VR40_Dro) for D-GABA_B_R3 that might form a functional heterodimer. We have determined the relationship of the receptors within subgroups of the large GPCR superfamily by means of a cross-genome phylogenetic clustering approach. These studies also revealed a higher-level phylogenetic organization in which clusters with common ligand structure or chemistry, or a shared function, are evident across genomes. We hope that this approach proves valuable for identifying the natural ligands of *Drosophila* and human orphan receptors.

**Table 2:** List of *Drosophila* orphan receptors

**Name**	**Swissprot Code**	**Best match receptor with known ligand; % Identity**	**Cluster**		**Description**
**Peptide receptors**					
Q8I943_Dro	Q8I943	SSR2_HUMAN; 40.2	2		Somatostatin receptor
Q8ISJ9_Dro	Q8ISJ9	SSR5_HUMAN; 45.8	2		Orphan GPCR
Q9V858_Dro	Q9V858	GRPR_HUMAN; 40.0	4		CG30106 protein
Q9V9K3_Dro	Q9V9K3	BRS3_HUMAN; 36.4	4		CG14593 protein
Q8SWR3_Dro	Q8SWR3		5		RE15519p; CG16752 protein
Q9V5T1_Dro	Q9V5T1	TRFR_HUMAN; 22.5	5		CG13229 protein; AT19640p
Q9VDC4_Dro	Q9VDC4	GHSR_HUMAN; 34.2	5		CG5911 protein
Q9VT27_Dro	Q9VT27	TRFR_HUMAN; 38.3	5		CG16726 protein
Q9W025_Dro	Q9W025	TRFR_HUMAN; 27.4	5		CG8985 protein
Q9W027_Dro	Q9W027	TRFR_HUMAN; 29.3	5		CG13803 protein
GRHRII_Dro	GRHRII_Dro	GRR2_HUMAN; 34.0	6		Putative corazonin receptor
Q8SX01_Dro	Q8SX01	LSHR_HUMAN; 50.0	7		RH44949p
Q9NDI1_Dro	Q9NDI1	LSHR_HUMAN; 48.9	7		Glycoprotein hormone receptor II
Q9VBP0_Dro	Q9VBP0	LGR8_HUMAN; 35.4	7		CG31096-PA
Q9VYG0_Dro	Q9VYG0	LGR8_HUMAN; 40.6	7		CG4187 protein
Q8MKU0_Dro	Q8MKU0	NFF2_HUMAN; 31.2	8		CG30340-PA
Q9VWQ9_Dro	Q9VWQ9	CCKR_HUMAN; 42.0	8		CG32540 protein
Q9VWR3_Dro	Q9VWR3	CCKR_HUMAN; 29.6	8		CG6857 protein
Q8SZ35_Dro	Q8SZ35	NY2R_HUMAN; 37.0	11		RE18294p
Q9VRM0_Dro	Q9VRM0	NYR_DROME; 37.9	11		CG10626 protein
Q9VW75_Dro	Q9VW75	NY1R_HUMAN; 36.6	11		CG7395 protein; GH23382p
Q9W189_Dro	Q9W189	NYR_DROME; 29.0	11		CG13575 protein
**Nucleotide and lipid receptors**				
Q9VTU7_Dro	Q9VTU7	OPS3_DROME; 38.6	14		CG5638 protein; GH14208p
Q9VVJ1_Dro	Q9VVJ1	O00325; 26.9	18		CG7497 protein; GH27361p
**Biogenic amine receptors**				
Q9VCZ3_Dro	Q9VCZ3	5H4_HUMAN; 44.3	20		CG6919 protein
Q9VG54_Dro	Q9VG54	5H4_HUMAN; 39.9	20		CG6989 protein
O77269_Dro	O77269	ML1A_HUMAN; 29.2	21		EG:22E5.10 protein
O77270_Dro	O77270	ML1A_HUMAN; 28.1	21		EG:22E5.11 protein
Q9VEG1_Dro	Q9VEG1	5H1A_HUMAN; 39.6	22		CG7431 protein
Q9VEG2_Dro	Q9VEG2	5HT1_DROME; 19.4	22		CG16766 protein
Q9VAA2_Dro	Q9VAA2	AA2A_HUMAN; 39.2	23		CG9753 protein
Q9VHW1_Dro	Q9VHW1	ACM3_HUMAN; 36.1	23	CG7918 protein
Q9VMI4_Dro	Q9VMI4	5HT1_DROME; 22.3	23		CG13995 protein; RE05601p
Q9VBG4_Dro	Q9VBG4	HH2R_HUMAN; 32.6	24		CG12290 protein; GH12381P
Q9VE32_Dro	Q9VE32	A2AA_HUMAN; 39.5	24		CG18208 protein
Q9W3V5_Dro	Q9W3V5	Q13675; 30.3	24		CG12796 protein
**Class B (secretin) receptors**				
Q9NEF7_Dro	Q9NEF7	CRF2_HUMAN; 37.0	25		EG:BACR25B3.3 protein
Q9V6C7_Dro	Q9V6C7	CRF2_HUMAN; 42.8	25		CG12370 protein
Q9V6N4_Dro	Q9V6N4	CGRR_HUMAN; 41.1	25		CG17043 protein
Q9V716_Dro	Q9V716	CRF2_HUMAN; 42.8	25		CG8422 protein
Q8INM0_Dro	Q8INM0	MTH_DROME; 38.2	26		CG31147-PA
Q8IPD0_Dro	Q8IPD0	MTHA_DROME; 29.8	26		CG31720-PB
**Cell adhesion receptors**				
Q8SZ78_Dro	Q8SZ78	CD97_HUMAN; 27.4	27		RE14222p
Q8T4B2_Dro	Q8T4B2	CD97_HUMAN; 26.6	27		AT07595p
Q9V4V8_Dro	Q9V4V8	CD97_HUMAN; 22.6	27		CG8639 protein
STAN_Dro	STAN_DROME	CD97_HUMAN; 50.8	27		Protocadherin-like wing polarity protein stan precursor; Starry night protein; Flamingo protein
**Class C (glutamate) receptors**				
Q9V4U4_Dro	Q9V4U4	Q8NFS4(MGR7_HUMAN); 41.1	28		CG30361 protein
BOSS_Dro	BOSS_DROME	O95357(RAIG1); 22.3	30		Bride of sevenless protein precursor
Q9VKA4_Dro	Q9VKA4	Q9Y133; 27.2	31		CG31760 protein
Q9VNZ5_Dro	Q9VNZ5	MGR_DROME; 32.3	31		CG32447 protein
Q9VR40_Dro	Q9VR40	GBR2_HUMAN; 31.4	31		CG31660 protein

## Methods

### Sequence data mining

Human (537) and *Drosophila* (284) GPCR amino acid sequences were downloaded from GPCRDB (7.0) [18]. The subset of entries containing the keyword 'olfactory receptors (OR)' or 'gustatory receptors (GR)' or 'taste receptors' were extracted by text parsing and were removed as they were extremely diverse sequences and inclusion of them affects badly on alignments quality. Further, we wanted to avoid polymorphism, splice variants, pseudogenes and duplicates of these receptors and sequences above 90% sequence identity were removed from the data set using CD-HIT [63]. This set amounted to 371 human and 113 *Drosophila* sequences (Additional data file 1). GPCRs without published ligands in the NCBI-PubMed  were considered as orphan receptors. The sequences were renamed to add suffix _Hum and _Dro to refer to human and *Drosophila* sequences respectively.

### Transmembrane helix predictions

Transmembrane domains were identified using HMMTOP program [64]. Amino termini upstream of TMH-1 and carboxyl termini downstream of TMH-7 were removed as they show extreme variability in these regions. Sequence comprising of TMH-1 to TMH-7 alone were considered for the analysis (Figure 2).

### Multiple sequence alignments

ClustalX 1.83 [65] was used for multiple sequence alignments (MSA) of receptors with a gap penalty of 10, a gap extension penalty of 0.05 and delay divergent sequences of 35% and protein weight matrix was BLOSUM series. The slow-accurate method was used for the initial pairwise alignments. The protein weight matrix was Blossom 30. When necessary, alignments were optimized by manual editing (Figure 2).

### Phylogenetic analysis

An overall phylogenetic tree was inferred from the multiple sequence alignment using PHYLIP package (V 3.5) [66]

### Sequence bootstrapping

The bootstrapping of multiple sequence alignment was performed 100 times using SEQBOOT to obtain 100 different alignments. Owing to the limitations in the CONSENSE program of Phylip package to handle large datasets, we restricted to 100 bootstrap replication steps [16].

### Neighbor-joining tree

Protein distances were calculated using PROTDIST from the PHYLIP package. The trees were calculated using Neighbor-Joining (NJ) method [67,68] on 100 different distance matrices using NEIGHBOR from the PHYLIP 3.5 package, resulting in 100 trees. These were analyzed using CONSENSE from the PHYLIP package to derive a bootstrapped consensus tree. An unrooted tree was plotted using TREEVIEW [69]. Sequences with more than 50% bootstrap support values were confirmed and grouped.

### Maximum likelihood trees

MSAs for each of the groups were obtained as described above and were used for building maximum likelihood trees [70] using TREE-PUZZLE 5.1 [71]. It is least affected by sampling errors and robust to many violations of the assumptions in the evolutionary model [72]. Parameters were estimated by Quartet sampling and NJ tree; The jones-taylor-thornton (JTT) substitution model was used for the calculation with amino acid usage estimated from data, site-to-site rate variation modeled on a gamma distribution with eight rate categories plus invariant sites, and the gamma distribution parameters estimated from the data. 10,000 quartet puzzling steps were performed to obtain support values for each internal branch and trees inferred with the highest likelihood. This method outperforms other methods like neighbor joining or parsimony methods except that it is computationally intensive, extremely slow and cannot be applied to very large datasets. *Drosophila* 5HTA receptor (5HTA_Dro) of family A was used as out-group for secretin, glutamate, cell adhesion and frizzled receptors. Human (O75205_Hum or GPRC5B) receptor of family B was used as out-group for peptide, chemokine, nucleotide and lipid and biogenic amine receptors for tree constructions (out-groups not shown in the figures) using Tree View [69].

### BLAST searches

For sequences with lower support values, similarity measures obtained by searching all against all sequences using BLASTP [73] were used to associate them to the clusters identified by PHYLIP and maximum likelihood methods. Manual inspection of the alignments, bit-score, E-Value, and length of pairwise alignments were considered as measures of similarity. Such receptors may be distantly related to members of the groups but may be sharing high structural similarity and common functional role, possibly due to convergent evolution [74]. It is also possible that these sequences are very diverse that the clustering methods were not sensitive enough to measure these changes [17].

## Authors' contributions

M.R.P. Rao has carried out the work and has written the first draft of the manuscript. R.S. has initiated the idea and was involved in discussions and drafting of the final manuscript.

## Supplementary Material

Additional data file 2 Key residues conserved among the members of cluster 17. Click here for file

Additional data file 1 Table indicating the cluster, accession numbers, swissprot codes, gene names and description of the GPCR sequences that have been used. Click here for file
